# Supplementary material for: Toward incompatible quantum limits on multiparameter estimation
Source: Nat Commun. 2023 Feb 23;14:1021. doi: 10.1038/s41467-023-36661-3 (PMC9950091; doi:10.1038/s41467-023-36661-3)
Supplement: Supplementary file 1 — Supplementary Information [file 41467_2023_36661_MOESM1_ESM.pdf]

Supplementary Information: Toward Incompatible Quantum Limits on Multiparameter Estimation

Binke Xia,[1] Jingzheng Huang,[1,2,3,*] Hongjing Li,[1,2,3] Han Wang,[1] and Guihua Zeng[1,2,3,†]

[1] *State Key Laboratory of Advanced Optical Communication Systems and Networks, Institute for Quantum Sensing and Information Processing, School of Sensing Science and Engineering, Shanghai Jiao Tong University, Shanghai 200240, China*

[2] *Hefei National Laboratory, Hefei 230088, China*

[3] *Shanghai Research Center for Quantum Sciences, Shanghai 201315, China*

* jzhuang1983@sjtu.edu.cn

† ghzeng@sjtu.edu.cn

Supplementary Note 1: Derivation of quantum multiparameter estimation criterion

In this section, we would like to give the derivation details of quantum multiparameter criterion (QMEC).

1.1. Trade-off precision relation of incompatible parameters

Recently, Xiaoming Lu and Xiaoguang Wang proposed a trade-off relation for the measurement inaccuracies of different parameters[1] by incorporating the Heisenberg's uncertainty principle (HUP) and Ozawa's uncertainty relation[2, 3]. Here we would like to begin with the measurement uncertainty relations. Let $\hat{A}$ and $\hat{B}$ be two Hermitian operators standing for the ideal observables we intend to measure. Due to the HUP, when $[\hat{A}, \hat{B}] \neq 0$, these two observables can not be jointly measured. To approximately the joint measurement of the non-commutative observables $\hat{A}$ and $\hat{B}$, we can dilate the system by adding an ancilla η, where another pair of commuting observables $\hat{\mathcal{A}}$ and $\hat{\mathcal{B}}$ acting on the dilated system are measured[4]. Then the measurement errors for the ideal observables $\hat{A}$ and $\hat{B}$ in the quantum state ρ is defined by Ozawa as:

$$\epsilon_A = \sqrt{\mathrm{tr}\left[\left(\hat{\mathcal{A}} - \hat{A} \otimes \hat{\mathbb{I}}\right)^2 (\rho \otimes \eta)\right]}, \quad \epsilon_B = \sqrt{\mathrm{tr}\left[\left(\hat{\mathcal{B}} - \hat{B} \otimes \hat{\mathbb{I}}\right)^2 (\rho \otimes \eta)\right]} \tag{1}$$

Combining with the uncertainties of observables $\hat{A}$ and $\hat{B}$ in the quantum state ρ:

$$\sigma_A = \sqrt{\mathrm{tr}\left(\hat{A}^2 \rho\right) - \mathrm{tr}\left(\hat{A}\rho\right)^2}, \quad \sigma_B = \sqrt{\mathrm{tr}\left(\hat{B}^2 \rho\right) - \mathrm{tr}\left(\hat{B}\rho\right)^2} \tag{2}$$

Ozawa derives the inequality:

$$\epsilon_A \epsilon_B + \epsilon_A \sigma_B + \epsilon_B \sigma_A \geq C_{AB} \equiv \frac{1}{2}\left|\mathrm{tr}\left([\hat{A}, \hat{B}]\rho\right)\right| \tag{3}$$

where $[\hat{A}, \hat{B}] \equiv \hat{A}\hat{B} - \hat{B}\hat{A}$, it is a universally joint-measurement error relation. Ozawa's inequality was recently strengthened by Branciard[3]:

$$\epsilon_A^2 \sigma_B^2 + \epsilon_B^2 \sigma_A^2 + 2\sqrt{\sigma_A^2 \sigma_B^2 - C_{AB}^2}\,\epsilon_A \epsilon_B \geq C_{AB}^2 \tag{4}$$

which is tight when ρ is a pure state.

Lu and Wang take $\hat{L}_i \to \hat{A}$ and $\hat{L}_j \to \hat{B}$, where $\hat{L}_i$ and $\hat{L}_j$ are respectively the symmetric logarithmic derivatives (SLD) for the parameters g_i and g_j in the parameterized quantum state $\rho_{\boldsymbol{g}}$[5], then the uncertainties can be calculated as:

$$\sigma_A \to \sqrt{\mathrm{tr}\left(\hat{L}_i^2 \rho_{\boldsymbol{g}}\right) - \mathrm{tr}\left(\hat{L}_i \rho_{\boldsymbol{g}}\right)^2} = \sqrt{\mathcal{Q}_{ii}}, \quad \sigma_B \to \sqrt{\mathrm{tr}\left(\hat{L}_j^2 \rho_{\boldsymbol{g}}\right) - \mathrm{tr}\left(\hat{L}_j \rho_{\boldsymbol{g}}\right)^2} = \sqrt{\mathcal{Q}_{jj}} \tag{5}$$

where $\mathcal{Q}_{ii}$ and $\mathcal{Q}_{jj}$ are respectively the i-th and j-th diagonal elements of quantum Fisher information matrix (QFIM) $\mathcal{Q}$. They also take $\hat{\mathcal{L}}_i \to \hat{\mathcal{A}}$ and $\hat{\mathcal{L}}_j \to \hat{\mathcal{B}}$, which are constructed from the SLD of the output state $\sum_\lambda \mathrm{tr}\left(\hat{\Pi}_\lambda \rho_{\boldsymbol{g}}\right) |\lambda\rangle\langle\lambda|$ under a set of POVM $\hat{\Pi} = \left\{\hat{\Pi}_\lambda \,|\, \hat{\Pi}_\lambda \geq 0, \sum_\lambda \hat{\Pi}_\lambda = \hat{\mathbb{I}}\right\}$, then the measurement errors ϵ_A and ϵ_B can be replaced by[1]:

$$\epsilon_A \to \sqrt{\mathcal{R}_{ii}} = \sqrt{\mathcal{Q}_{ii} - \mathcal{F}_{ii}}, \quad \epsilon_B \to \sqrt{\mathcal{R}_{jj}} = \sqrt{\mathcal{Q}_{jj} - \mathcal{F}_{jj}} \tag{6}$$

where $\mathcal{F}_{jj}$ is the classical Fisher information matrix under the given POVM $\hat{\Pi}$, and $\mathcal{R} = \mathcal{Q} - \mathcal{F}$ is named as information regret. Substituting Eq. (5) and Eq. (6) into the Branciard's inequality, Lu and Wang derive the trade-off relation between the information regrets of different parameters[1]:

$$\mathcal{R}_{ii}\mathcal{Q}_{jj} + \mathcal{R}_{jj}\mathcal{Q}_{ii} + 2\sqrt{\mathcal{Q}_{ii}\mathcal{Q}_{jj} - C_{ij}^2}\sqrt{\mathcal{R}_{ii}\mathcal{R}_{jj}} \geq C_{ij}^2 \tag{7}$$

where $C_{ij} \equiv \frac{1}{2}\left|\mathrm{tr}\left([\hat{L}_i, \hat{L}_j]\rho_{\boldsymbol{g}}\right)\right|$. This trade-off relation is tight for pure state $\rho_{\boldsymbol{g}} = |\psi_{\boldsymbol{g}}\rangle\langle\psi_{\boldsymbol{g}}|$. For pure state, it is easy to derive that $C_{ij} = 2\mathcal{C}_{ij}$, where $\mathcal{C}$ is the Berry curvature (BC). Furthermore, combining with the classical Cramér-Rao

inequalities $(\delta g_i)^2 \geq 1/(\nu \mathcal{F}_{ii})$ and $(\delta g_j)^2 \geq 1/(\nu \mathcal{F}_{jj})$, the trade-off relation can be expressed as:

$$2 - \frac{1}{\nu\,(\delta \tilde{g}_i)^2} - \frac{1}{\nu\,(\delta \tilde{g}_j)^2} + 2\sqrt{1 - \frac{4\mathcal{C}_{ij}^2}{\mathcal{Q}_{ii}\mathcal{Q}_{jj}}}\sqrt{\left[1 - \frac{1}{\nu\,(\delta \tilde{g}_i)^2}\right]\left[1 - \frac{1}{\nu\,(\delta \tilde{g}_j)^2}\right]} \geq \frac{4\mathcal{C}_{ij}^2}{\mathcal{Q}_{ii}\mathcal{Q}_{jj}} \tag{8}$$

where ν is the measured samples number.

1.2. QMEC of general quantum metrological process

Based on the trade-off relation in Eq. (8), it is easy to determine that the QMEC is given by:

$$\mathcal{S}_{ij} = \frac{\mathcal{Q}_{ii}\mathcal{Q}_{jj}}{4\mathcal{C}_{ij}^2} = \frac{\mathrm{Re}\mathcal{T}_{ii}\mathrm{Re}\mathcal{T}_{jj}}{(\mathrm{Im}\mathcal{T}_{ij})^2} \tag{9}$$

where $\mathcal{T}$ is the quantum geometric tensor (QGT), which can be calculated as:

$$\begin{aligned}\mathcal{T}_{ij} \quad &= \frac{\partial \langle \psi_{\boldsymbol{g}}|}{\partial g_i}\left(1 - |\psi_{\boldsymbol{g}}\rangle\langle\psi_{\boldsymbol{g}}|\right)\frac{\partial |\psi_{\boldsymbol{g}}\rangle}{\partial g_j} \\ &= \langle\psi|\frac{\partial \hat{U}^\dagger}{\partial g_i}\left(1 - \hat{U}|\psi\rangle\langle\psi|\hat{U}^\dagger\right)\frac{\partial \hat{U}}{\partial g_j}|\psi\rangle \\ &= \langle \hat{H}_i\hat{H}_j\rangle - \langle \hat{H}_i\rangle\langle \hat{H}_j\rangle\end{aligned} \tag{10}$$

where $\hat{H}_i = \mathrm{i}\left[\frac{\partial}{\partial g_i}\hat{U}^\dagger(\boldsymbol{g})\right]\hat{U}(\boldsymbol{g})$ is the generator of parameter g_i, and $\langle\cdot\rangle$ denotes for $\langle\psi|\cdot|\psi\rangle$. Then the QFIM and BC can be respectively given by:

$$\mathcal{Q}_{ij} = 4\mathrm{Re}\mathcal{T}_{ij} = 2\langle\{\hat{H}_i, \hat{H}_j\}\rangle - 4\langle\hat{H}_i\rangle\langle\hat{H}_j\rangle = 4\mathrm{Cov}_{|\psi\rangle}\left(\hat{H}_i, \hat{H}_j\right) \tag{11}$$

where $\{\hat{H}_i, \hat{H}_j\} = \hat{H}_i\hat{H}_j + \hat{H}_j\hat{H}_i$, and

$$\mathcal{C}_{ij} = -2\mathrm{Im}\mathcal{T}_{ij} = \mathrm{i}\langle[\hat{H}_i, \hat{H}_j]\rangle \tag{12}$$

Therefore, the QMEC can be calculated by:

$$\mathcal{S}_{ij} = \frac{\mathcal{Q}_{ii}\mathcal{Q}_{jj}}{4\mathcal{C}_{ij}^2} = \frac{4\langle\Delta\hat{H}_i^2\rangle\langle\Delta\hat{H}_j^2\rangle}{\left|\langle[\hat{H}_i, \hat{H}_j]\rangle\right|^2} \tag{13}$$

where $\langle\Delta\hat{H}_i\rangle = \langle\hat{H}_i^2\rangle - \langle\hat{H}_i\rangle^2$ and $\langle\Delta\hat{H}_j\rangle = \langle\hat{H}_j^2\rangle - \langle\hat{H}_j\rangle^2$.

1.3. QMEC of post-selected weak measurement scheme

In the multiparameter weak measurement scheme, every unknown parameter $g_j \in \boldsymbol{g} = (g_1, g_2, \ldots, g_n)$ is coupled to the pointer via a corresponding translation operator $\hat{\Omega}_j$ during the weak interaction procedure[6]. Then the weak interaction process is described by an impulse Hamiltonian:

$$\hat{H}_{\mathrm{I}} = \delta\,(t - t_0)\sum_{j=1}^{n} g_j\hat{A}\otimes\hat{\Omega}_j \tag{14}$$

where $\hat{A}$ is an hermitian operator on the two-level system. Then the evolution operator in multiparameter weak measurement scheme is

$$\hat{U}_{\mathrm{w}} = \exp\left(-\mathrm{i}\int \hat{H}_{\mathrm{I}}\mathrm{d}t\right) \approx 1 - \mathrm{i}\sum_{j=1}^{n} g_j\hat{A}\otimes\hat{\Omega}_j \tag{15}$$

The initial state of the whole system is denoted as $|\Psi_i\rangle = |i\rangle|\psi_i\rangle$, where $|i\rangle$ is the pre-selected state of the two-level system, and $|\psi_i\rangle$ is the initial state of pointer. After the weak interaction process, the two-level system is post-selected by state $|f\rangle$, then the final state of the whole system is

$$\begin{aligned} |\Psi_f\rangle &= |f\rangle\langle f|\hat{U}_{\mathrm{w}}|\Psi_i\rangle \approx |f\rangle\langle f| \left(1 - \mathrm{i}\sum_{j=1}^{n} g_j \hat{A}\otimes\hat{\Omega}_j\right)|i\rangle|\psi_i\rangle \\ &= \langle f|i\rangle \left(1 - \mathrm{i}A_w \sum_{j=1}^{n} g_j \hat{\Omega}_j\right)|\psi_i\rangle|f\rangle = \langle f|i\rangle|\widetilde{\psi}_f\rangle|f\rangle \end{aligned} \tag{16}$$

where $A_w = \langle f|\hat{A}|i\rangle/\langle f|i\rangle$ is the weak value, and $|\widetilde{\psi}_f\rangle$ is the final state of pointer. Here, we we calculate the approximate results based on the weak interaction condition: $g_j \ll 1$, $j = 1, 2, \ldots, n$, and only the first-order small quantity is retained. Then the final pointer state $|\widetilde{\psi}_f\rangle$ can be normalized by a normalization factor:

$$\mathcal{N} = \frac{1}{\sqrt{\langle\widetilde{\psi}_f|\widetilde{\psi}_f\rangle}} = \frac{1}{\sqrt{1 + 2\left(\mathrm{Im}A_w\right)\sum_{j=1}^{n} g_j\langle\hat{\Omega}_j\rangle_i + \left|A_w\right|^2 \sum_{j=1}^{n}\sum_{l=1}^{n} g_j g_l \langle\hat{\Omega}_j\hat{\Omega}_l\rangle_i}} \approx 1 - \mathrm{Im}A_w \sum_{j=1}^{n} g_j\langle\hat{\Omega}_j\rangle_i \tag{17}$$

where $\langle\cdot\rangle_i$ denotes for $\langle\psi_i|\cdot|\psi_i\rangle$, the normalized final pointer state is:

$$|\psi_f\rangle = \mathcal{N}\left(1 - \mathrm{i}A_w \sum_{j=1}^{n} g_j\hat{\Omega}_j\right)|\psi_i\rangle \tag{18}$$

Thus, it is easy to determine that the QGT in multiparameter weak measurement scheme is:

$$\begin{aligned} \mathcal{T}_{jl} &= \frac{\partial\langle\psi_f|}{\partial g_j}\left(1 - |\psi_f\rangle\langle\psi_f|\right)\frac{\partial|\psi_f\rangle}{\partial g_l} \\ &\approx \mathcal{N}^2 \left|A_w\right|^2 \langle\hat{\Omega}_j\hat{\Omega}_l\rangle_i - \mathcal{N}^4 \left|A_w\right|^2 \langle\hat{\Omega}_j\rangle_i\langle\hat{\Omega}_l\rangle_i \\ &\approx \left|A_w\right|^2 \langle\hat{\Omega}_j\hat{\Omega}_l\rangle_i - \left|A_w\right|^2 \langle\hat{\Omega}_j\rangle_i\langle\hat{\Omega}_l\rangle_i \end{aligned} \tag{19}$$

The corresponding QFIM and BC is then calculated as:

$$\mathcal{Q}_{jl} = 4\mathrm{Re}\mathcal{T}_{jl} = 2\left|A_w\right|^2 \langle\{\hat{\Omega}_j, \hat{\Omega}_l\}\rangle_i - 4\left|A_w\right|^2 \langle\hat{\Omega}_j\rangle_i\langle\hat{\Omega}_l\rangle_i = 4\left|A_w\right|^2 \mathrm{Cov}_{|\psi_i\rangle}\left(\hat{\Omega}_j, \hat{\Omega}_l\right) \tag{20}$$

and

$$\mathcal{C}_{jl} = -2\mathrm{Im}\mathcal{T}_{jl} = \mathrm{i}\left|A_w\right|^2 \langle[\hat{\Omega}_j, \hat{\Omega}_l]\rangle_i \tag{21}$$

Then the QMEC of post-selected weak measurement scheme can be calculated as:

$$\mathcal{S}_{jl} = \frac{\mathcal{Q}_{jj}\mathcal{Q}_{ll}}{4\mathcal{C}_{jl}^2} = \frac{4\langle\Delta\hat{\Omega}_j^2\rangle_i\langle\Delta\hat{\Omega}_l^2\rangle_i}{\left|\langle[\hat{\Omega}_j, \hat{\Omega}_l]\rangle_i\right|^2} \tag{22}$$

Moreover, we can approximately take $|\psi_f\rangle \approx \left(1 - \mathrm{i}A_w \sum_{j=1}^{n} g_j\hat{\Omega}_j\right)|\psi_i\rangle$ in this case. In this work, we concentrate on the incompatible parameters generated by operators $\hat{P}$ and $\hat{X}$, then the corresponding QMEC can be calculated as:

$$\mathcal{S}_{12} = \mathcal{S}_{21} = 4\langle\Delta\hat{P}^2\rangle_i\langle\Delta\hat{X}^2\rangle_i \tag{23}$$

Supplementary Note 2: Simultaneous quantum limits and energy level of probe state

In this section, we would like to clarify that the attainability of simultaneous quantum limits for incompatible parameters is independent of the energy level of initial probe state, where two situations are involved: (1) two Gaussian states with different energy levels; and (2) Gaussian state and n-order HG state with the same energy level. Here, we

still concern with the simultaneous measurement of incompatible parameters g_1 and g_2 generated by the momentum operator $\hat{P}$ and the position operator $\hat{X}$ where the unitary parameterization is $\hat{U}(\boldsymbol{g}) = \exp\left(-\mathrm{i}g_1\hat{P} - \mathrm{i}g_2\hat{X}\right)$. First, considering the time-independent solution $\psi_n(x,\omega)$ for the Schrödinger equation of harmonic oscillators:

$$\mathrm{i}\hbar\frac{\partial}{\partial t}\psi = \left(\frac{\hat{P}^2}{2m} + \frac{1}{2}m\omega^2\hat{X}^2\right)\psi \tag{24}$$

For energy level $E_n = \left(n+\frac{1}{2}\right)\hbar\omega$, the corresponding eigenket is $|n,\omega\rangle = \int \mathrm{d}x\,\psi_n(x,\omega)|x\rangle$, which corresponds to the n-order HG state. And $|0,\omega\rangle$ corresponds to the Gaussian state with energy level $E_n = \frac{1}{2}\hbar\omega$. It is easy to obtain that:

$$\langle\Delta\hat{P}^2\rangle\Big|_{|n,\omega\rangle} = \langle n,\omega|\hat{P}^2|n,\omega\rangle - \langle n,\omega|\hat{P}|n,\omega\rangle^2 = \frac{\hbar}{2}(2n+1)m\omega \tag{25}$$

$$\langle\Delta\hat{X}^2\rangle\Big|_{|n,\omega\rangle} = \langle n,\omega|\hat{X}^2|n,\omega\rangle - \langle n,\omega|\hat{X}|n,\omega\rangle^2 = \frac{\hbar}{2}\frac{2n+1}{m\omega} \tag{26}$$

$$\langle[\hat{P},\hat{X}]\rangle\Big|_{|n,\omega\rangle} = \langle n,\omega|[\hat{P},\hat{X}]|n,\omega\rangle = -\mathrm{i}\hbar \tag{27}$$

2.1. Two Gaussian states with different energy levels

Denoting the initial probe state $|\psi_1\rangle = |0,\omega_1\rangle$, and the initial probe state $|\psi_2\rangle = |0,\omega_2\rangle$, where $\omega_1 < \omega_2$. Thus, the energy level of state $|\psi_1\rangle$ ($E^{(1)} = \frac{1}{2}\hbar\omega_1$) is smaller than the energy level of state $|\psi_2\rangle$ ($E^{(2)} = \frac{1}{2}\hbar\omega_2$). Performing the unitary parameterization $\hat{U}(\boldsymbol{g}) = \exp\left(-\mathrm{i}g_1\hat{P} - \mathrm{i}g_2\hat{X}\right)$ on state $|\psi_1\rangle$, the corresponding QFIM regarding the parameters g_1 and g_2 can be calculated as:

$$\mathcal{Q}(\boldsymbol{g},|0,\omega_1\rangle) = \begin{pmatrix} 2\hbar m\omega_1 & 0 \\ 0 & \frac{2\hbar}{m\omega_1} \end{pmatrix} \tag{28}$$

Similarly, performing the unitary parameterization $\hat{U}(\boldsymbol{g}) = \exp\left(-\mathrm{i}g_1\hat{P} - \mathrm{i}g_2\hat{X}\right)$ on state $|\psi_2\rangle$, the corresponding QFIM regarding the parameters g_1 and g_2 can be calculated as:

$$\mathcal{Q}(\boldsymbol{g},|0,\omega_2\rangle) = \begin{pmatrix} 2\hbar m\omega_2 & 0 \\ 0 & \frac{2\hbar}{m\omega_2} \end{pmatrix} \tag{29}$$

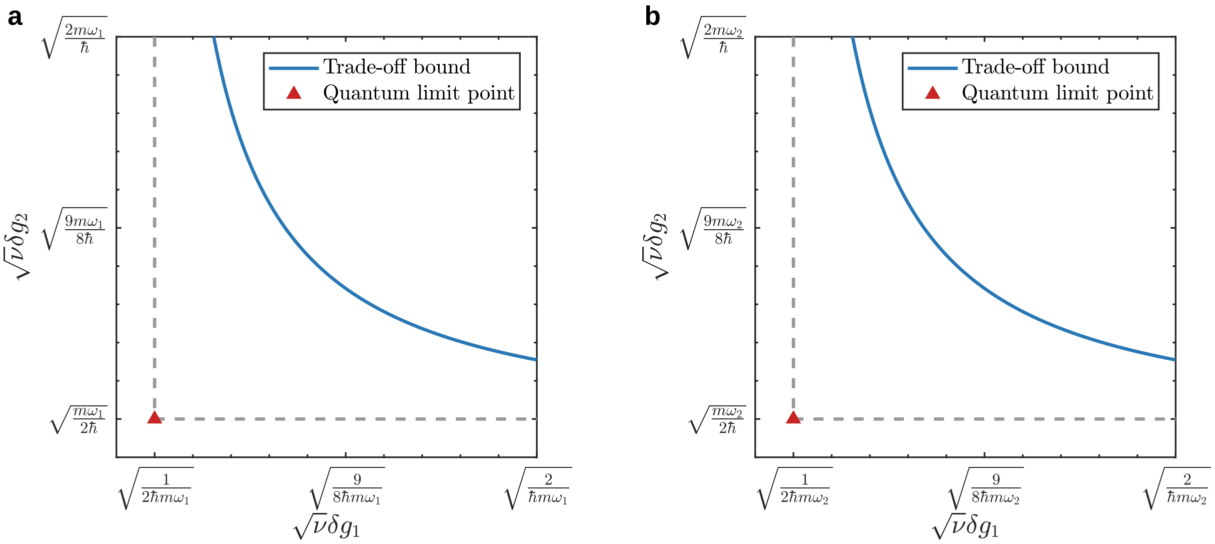

Supplementary Figure 1. Trade-off bounds of two Gaussian states with different energy levels. **a** Gaussian state with energy level $\frac{1}{2}\hbar\omega_1$. **b** Gaussian state with energy level $\frac{1}{2}\hbar\omega_2$.

It is easy to determine that $\mathcal{Q}(g_1, |0,\omega_1\rangle) < \mathcal{Q}(g_1, |0,\omega_2\rangle)$, but $\mathcal{Q}(g_2, |0,\omega_1\rangle) > \mathcal{Q}(g_2, |0,\omega_2\rangle)$, which means that increasing the energy level of Gaussian state will improve the quantum precision limit of parameter g_1, but reduce the quantum precision limit of parameter g_2. However, the attainability of simultaneous quantum limits for parameters g_1 and g_2 depends on the QMEC $\mathcal{S}_{12}$, where the Gaussian states $|0,\omega_1\rangle$ and $|0,\omega_2\rangle$ give the same results:

$$\mathcal{S}_{12}(|0,\omega_1\rangle) = \mathcal{S}_{12}(|0,\omega_2\rangle) = 1 \tag{30}$$

Thus, the curve properties of the trade-off bounds with the Gaussian states $|0,\omega_1\rangle$ and $|0,\omega_2\rangle$ are the same. This result leads to the same attainability of simultaneous quantum limits for parameters g_1 and g_2 with the Gaussian states $|0,\omega_1\rangle$ and $|0,\omega_2\rangle$. For illustration, we have plotted the trade-off bounds with the Gaussian states $|0,\omega_1\rangle$ and $|0,\omega_2\rangle$ in **Supplementary Figure 1**.

2.2. *Gaussian state and n-order HG state with the same energy level*

Denoting the initial probe state $|\psi_1\rangle = |0,\omega_1\rangle$, and the initial probe state $|\psi_2\rangle = |n,\omega_2\rangle$, where $\omega_1 = (2n+1)\omega_2$. Thus, the energy level of state $|\psi_1\rangle$ ($E^{(1)} = \frac{1}{2}\hbar\omega_1$) is the same as that of state $|\psi_2\rangle$ ($E^{(2)} = \left(n+\frac{1}{2}\right)\frac{1}{2}\hbar\omega_2 = \frac{1}{2}\hbar\omega_1$). Performing the unitary parameterization $\hat{U}(\boldsymbol{g}) = \exp\left(-\mathrm{i}g_1\hat{P} - \mathrm{i}g_2\hat{X}\right)$ on state $|\psi_1\rangle$, the corresponding QFIM regarding the parameters g_1 and g_2 can be calculated as:

$$\mathcal{Q}(\boldsymbol{g}, |0,\omega_1\rangle) = \begin{pmatrix} 2\hbar m\omega_1 & 0 \\ 0 & \frac{2\hbar}{m\omega_1} \end{pmatrix} \tag{31}$$

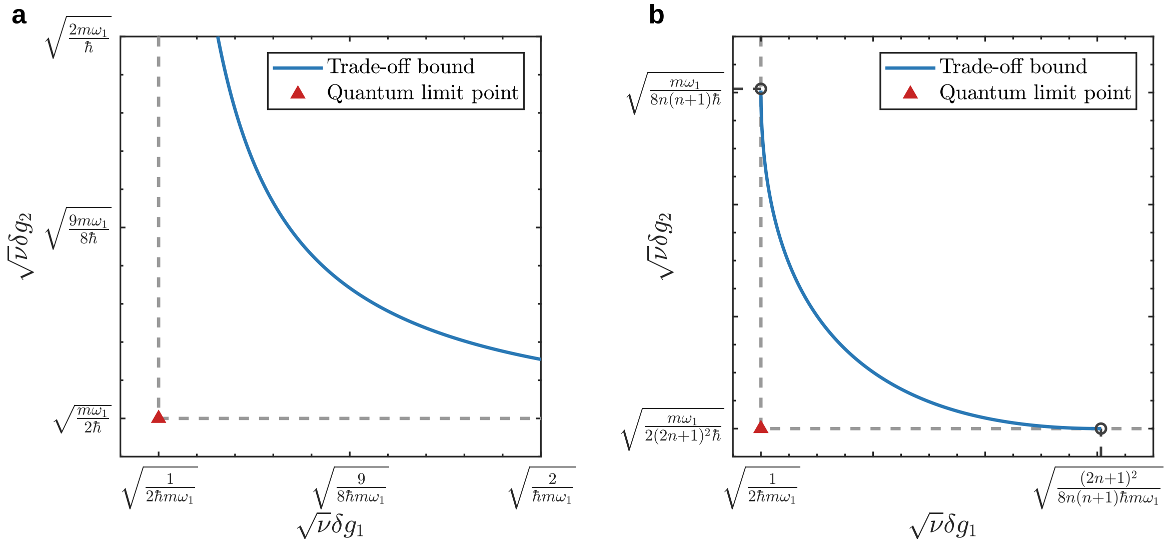

Supplementary Figure 2. Trade-off bounds of a Gaussian state and a HG$_n$ state on the same energy level. **a** Gaussian state with energy level $\frac{1}{2}\hbar\omega_1$. **b** HG$_n$ state with energy level $(n+\frac{1}{2})\hbar\omega_2 = \frac{1}{2}\hbar\omega_1$.

Similarly, performing the unitary parameterization $\hat{U}(\boldsymbol{g}) = \exp\left(-\mathrm{i}g_1\hat{P} - \mathrm{i}g_2\hat{X}\right)$ on state $|\psi_2\rangle$, the corresponding QFIM regarding the parameters g_1 and g_2 can be calculated as:

$$\mathcal{Q}(\boldsymbol{g}, |n,\omega_2\rangle) = (2n+1)\begin{pmatrix} 2\hbar m\omega_2 & 0 \\ 0 & \frac{2\hbar}{m\omega_2} \end{pmatrix} = \begin{pmatrix} 2\hbar m\omega_1 & 0 \\ 0 & \frac{2(2n+1)^2\hbar}{m\omega_1} \end{pmatrix} \tag{32}$$

It is easy to determine that $\mathcal{Q}(g_1, |0,\omega_1\rangle) = \mathcal{Q}(g_1, |n,\omega_2\rangle)$, but $\mathcal{Q}(g_2, |0,\omega_1\rangle) = \frac{1}{(2n+1)^2}\mathcal{Q}(g_2, |n,\omega_2\rangle)$. Therefore, giving the Gaussian state and the HG$_n$ state on the same energy level, the quantum limits for the Gaussian state $|0,\omega_1\rangle$ and the HG$_n$ state $|n,\omega_2\rangle$ are the same regarding the parameter g_1, but the quantum limit for the HG$_n$ state $|n,\omega_2\rangle$ is $(2n+1)^2$-fold more precise than the Gaussian state $|0,\omega_1\rangle$ regarding the parameter g_2.

Although the Gaussian state and the HG$_n$ state are on the same energy level, their corresponding QMECs are difference, which are calculated as:

$$\mathcal{S}_{12}(|0,\omega_1\rangle) = 1, \qquad \mathcal{S}_{12}(|n,\omega_2\rangle) = \frac{1}{(2n+1)^2} \tag{33}$$

Thus, the attainability of simultaneous quantum limits for the incompatible parameters g_1 and g_2 with the HG$_n$ state $|n,\omega_2\rangle$ is improved $(2n+1)^2$-fold than that with the Gaussian state $|0,\omega_1\rangle$, though they are on the same energy level. For illustration, we have plotted the trade-off bounds with the Gaussian states $|0,\omega_1\rangle$ and the HG$_n$ state $|n,\omega_2\rangle$ separately in **Supplementary Figure 2**.

From these two examples, we can conclude that the QMEC $\mathcal{S}_{12}$ for evaluating the attainability of simultaneous quantum limits is independent of the energy level of initial probe state. The attainability of simultaneous quantum limits depends on the uncertainty properties of the initial probe state regarding the parameters' generators.

Supplementary Note 3: Details of experimental set-up

3.1. Generation method of HG beams

Traditionally, mode cleaner cavity is necessary for generating high-order HG beams[7, 8]. However, mode cleaner cavity is usually difficult to setup and control in experiment. In this work, we generate the high-order HG beams by a SLM and 4-f spatial filter system[9], which is easier to implement in experiment. And the generation scheme is illustrated in **Supplementary Figure 3**.

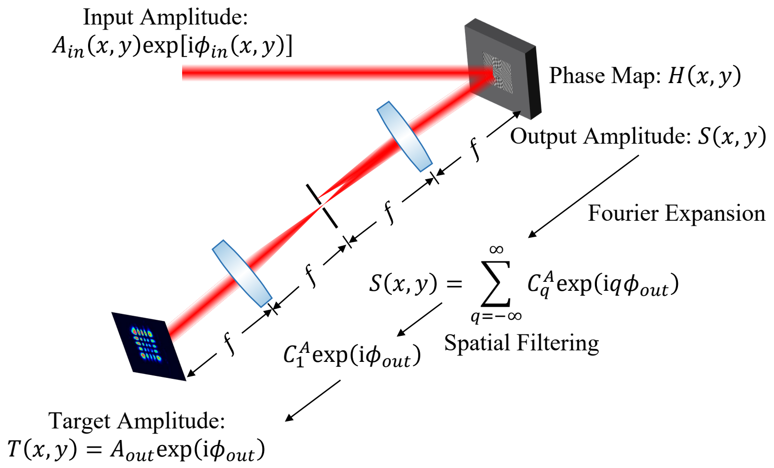

Supplementary Figure 3. Generation scheme of high-order HG beams. The expanded Gaussian beam is inputted into the spatial light modulator (SLM), and a 4-f spatial filter system is used to choose the expected mode.

In this scheme, the light beam from a 780 nm DBR laser was expanded to a 8.6 mm-width Gaussian beam by a fiber coupler. The complex amplitude of expanded Gaussian beam is denoted as: $A_{in}(x,y)\exp[\mathrm{i}\phi_{in}(x,y)]$. Then inputting this light into a SLM, where the phase map $H(x,y)$ is displayed. The output amplitude of SLM can be denoted as:

$$S(x,y) = A_{in}(x,y)\exp[\mathrm{i}\phi_{in}(x,y) + \mathrm{i}H(x,y)] \tag{34}$$

Here, we denote the relative phase as: $\phi_r = \phi_{out} - \phi_{in} + \phi_g$, where ϕ_g is the grating phase, and the relative amplitude is $A_r = A_{out}/A_{in}$. To filter the target light, we let

$$H(x,y) = f(A_r)\sin(\phi_r) \tag{35}$$

Based on the Bessel expansion formula:

$$\exp[\mathrm{i}f(a)\sin(\phi)] = \sum_{-\infty}^{\infty} J_q[f(a)]\exp(iq\phi) \tag{36}$$

where $J_q[\cdot]$ is the q-order Bessel function. Thus, we have the amplitude of the 1st-order diffraction beam is $A_{in} \cdot J_1[f(A_r)]\exp(i\phi_{out})$. The mapping function $f(\cdot)$ can be easily derived as:

$$f(A_r) = J_1^{-1}(A_r) \tag{37}$$

where $J_1^{-1}(A_r)$ is the inverse function of 1st-order Bessel function. By employing a 4-f spatial filter system with an aperture at the 1st-order diffraction point, we can generate any target beam amplitude $T(x,y) = A_{out}\exp(i\phi_{out})$ with the displayed phase map $H(x,y) = J_1^{-1}(A_r)\sin(\phi_r)$ on SLM.

3.2. Phase lock in the interferometer

In our experiment, the incompatible parameters were simultaneously generated in a polarized Mach-Zehnder interferometer (MZI). To stabilize the relative phase of two optical path in the MZI, a lock-in amplifier was employed, as is illustrated in **Supplementary Figure 4**.

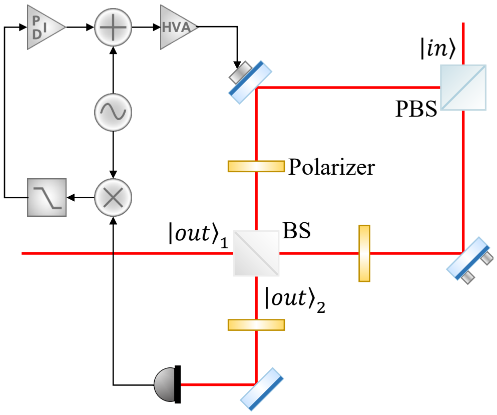

Supplementary Figure 4. Schematic of lock-in amplifier in the MZI. The second output port of the MZI is used to monitor the relative phase of the interferometer. The error signal is feedback to the PZT driven mirror to control the relative phase in the interferometer.

Here, we would like to briefly introduce how to lock the relative phase in our polarized MZI. The input polarization state is denoted as $|in\rangle = \frac{1}{\sqrt{2}}(|H\rangle + |V\rangle)$. We exerted a cosine modulation $\beta\cos(2\pi ft)$ on the $|V\rangle$ path in the MZI via a PZT driving mirror, where β is the modulation depth. Denoting the relative phase in the two optical path as ϕ, then the two output polarization states are separately calculated as:

$$\begin{aligned} |out\rangle_1 &= \frac{1}{\sqrt{2}}\left(|H\rangle + \mathrm{e}^{\mathrm{i}\phi+\mathrm{i}\beta\cos(2\pi ft)}|V\rangle\right) \\ |out\rangle_2 &= \frac{1}{\sqrt{2}}\left(|H\rangle - \mathrm{e}^{\mathrm{i}\phi+\mathrm{i}\beta\cos(2\pi ft)}|V\rangle\right) \end{aligned} \tag{38}$$

The output state $|out\rangle_2$ was projected to the polarization state $|+\rangle = \frac{1}{\sqrt{2}}(|H\rangle + |V\rangle)$ via a polarizer, and then detected by a photodetector (PD) with probability:

$$\begin{aligned} P_{\mathrm{det}} &= |\langle +|out\rangle_2|^2 = \frac{1}{4}\left|1 - \mathrm{e}^{\mathrm{i}\phi+\mathrm{i}\beta\cos(2\pi ft)}\right|^2 = \frac{1}{2} - \frac{1}{2}\mathrm{Re}\left[\mathrm{e}^{\mathrm{i}\phi+\mathrm{i}\beta\cos(2\pi ft)}\right] \\ &= \frac{1}{2}\left[1 + J_0(\beta)\cos\phi\right] + \sin\phi\sum_{k=1}^{\infty} J_{2k-1}(\beta)\cos\left[2\pi(2k-1)ft\right] + \cos\phi\sum_{k=1}^{\infty} J_{2k}(\beta)\cos\left[2\pi(2kf)t\right] \end{aligned} \tag{39}$$

The detected optical power in PD $I_{\mathrm{det}} \propto P_{\mathrm{det}}$, then the detected power at frequency f is $I_{\mathrm{det}}^{(f)} \propto J_1(\beta)\sin\phi$. In the lock-in amplifier, the detected optical signal was demodulated by multiplying a cosine signal at frequency f, and then a low-pass filter was employed to get the signal $I_{\mathrm{det}}^{(f)}$. Finally, a PID controller and a high-voltage amplifier (HVA) were used to feed bake the error signal to the PZT driving mirror for stabilizing the relative phase $\phi = 0$.

In **Supplementary Figure 5**, we illustrated the detected ellipticities of polarization state $|out\rangle_1$. The orange line stands for the ellipticity of output polarization with lock-in amplifier, and the blue line stands for the ellipticity of

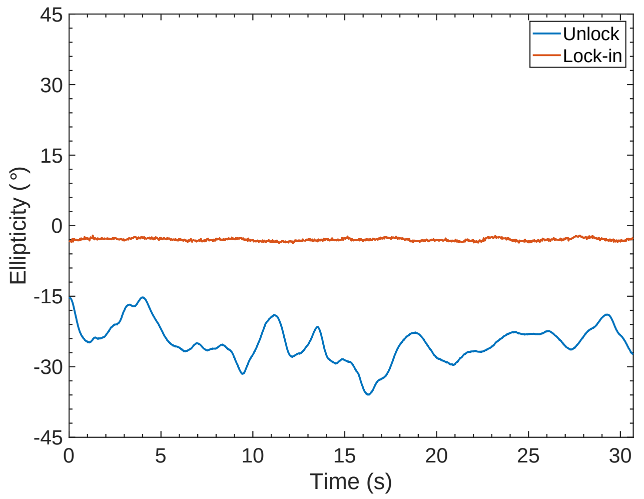

Supplementary Figure 5. Ellipticities of the output polarization with and without lock-in amplifier. The orange line is the polarization ellipticity of the output light of the MZI with phase lock-in. The blue line is the polarization ellipticity of the output light of the MZI with phase unlocked.

output polarization without lock-in amplifier. Obviously, the relative phase was well stabilized in our interferometer with lock-in amplifier.

3.3. Projective measurement

In our experimental scheme, the rotation signal was finally detected by projective measurement[10], which is illustrated in **Supplementary Figure 6**. Suppose that the input light field on SLM is $g(x, y)$, and the modulation light field on SLM is $h(x, y)$ (modulation method is same as the generation method of HG beams). The input field and the

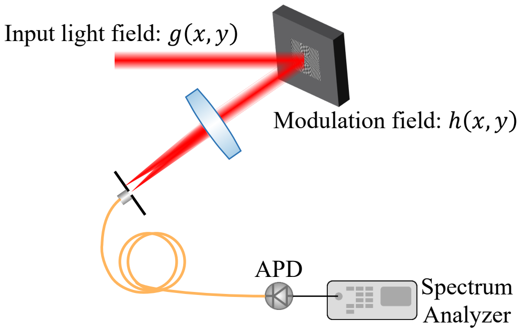

Supplementary Figure 6. Projective measurement scheme. The input light field is modulated by the SLM, and a single mode fiber (SMF) is used to filter the expected light filed. Then the detected light power in the APD is proportional to the projective probability.

modulation field are simply combined as $g(x, y)h(x, y)$ on SLM, and a Fourier lens transfer this filed to

$$f(u, v) = \mathscr{F}\left[g(x, y)h(x, y)\right] = \iint_{-\infty}^{+\infty} g(x, y)h(x, y)\exp\left[-i2\pi(xu + yv)\right]\mathrm{d}x\mathrm{d}y \tag{40}$$

which is spatially filtered by a SMF coupled to an APD, the coupling efficiency into the fiber is given as:

$$\eta \propto \left| \iint_{-\infty}^{+\infty} f(u, v)\exp\left(\frac{u^2 + v^2}{w_f^2}\right)\mathrm{d}u\mathrm{d}v \right|^2 \tag{41}$$

where w_f is the field width of fiber mode. In our experiment, $w_f = 4.6\,\mu\mathrm{m}$, which is much smaller of size scale than the features of $f(u, v)$. (The focal length of the Fourier lens is 10 cm, which transfer the waist width of 500 µm HG00

beam to nearly 50 µm.) Therefore, we have $\iint f(u,v)\exp\left[(u^2+v^2)/w_f^2\right]\mathrm{d}u\mathrm{d}v \approx \iint f(0,0)\exp\left[(u^2+v^2)/w_f^2\right]\mathrm{d}u\mathrm{d}v$, which leads to

$$\eta \propto |f(0,0)|^2 = \left|\iint_{-\infty}^{+\infty} g(x,y)h(x,y)dxdy\right|^2 = |\langle h^*|g\rangle|^2 \tag{42}$$

In this experiment, we implemented the projection measurement $\hat{\Pi}_1 = |u_{\hat{X}}^{\perp}\rangle\langle u_{\hat{X}}^{\perp}|$ by displaying the phase map of spatial function

$$u_{\hat{X}}^{\perp}(x) = \frac{1}{\sqrt{2n+1}}\left[\sqrt{n+1}u_{n-1}(x,z_0) - \sqrt{n}u_{n+1}(x,z_0)\right] \tag{43}$$

on the SLM. Similarly, the projection measurement $\hat{\Pi}_2 = |u_{\hat{P}}^{\perp}\rangle\langle u_{\hat{P}}^{\perp}|$ was implemented by displaying the phase map of spatial function

$$u_{\hat{P}}^{\perp}(x) = \frac{1}{\sqrt{2n+1}}\left[\sqrt{n+1}u_{n-1}(x,z_0) + \sqrt{n}u_{n+1}(x,z_0)\right] \tag{44}$$

on the SLM.

Supplementary Note 4: Supplement of experimental results

In practice, the amplitudes of displacement and tilt signals are reflected by the levels of the peak power at 2 kHz on the spectrum analyzer. When displaying $\hat{\Pi}_i$ $(i=1,2)$ projection measurement on the SLM, the practical detected signal-to-noise ratio in the experiment is obtained from:

$$\mathrm{SNR}_i = \frac{I_i^{(2\,\mathrm{kHz})}}{\delta I_i} = \frac{V_{\mathrm{s}i}}{V_{\mathrm{sn}i}} = \frac{V_{\mathrm{p}i} - V_{\mathrm{n}i}}{V_{\mathrm{n}i} - V_{\mathrm{en}}} \tag{45}$$

where $V_{\mathrm{sn}i} \propto \delta I_i$ is the level of detected shot-noise floor and $V_{\mathrm{s}i} \propto I_i^{(2\,\mathrm{kHz})}$ is the level of signal at 2 kHz. The shot-noise level is calculated by $V_{\mathrm{sn}i} = V_{\mathrm{n}i} - V_{\mathrm{en}}$, where $V_{\mathrm{n}i}$ is the level of total noise floor on the spectrum analyzer, and V_{en} is the level of electrical noise floor on the spectrum analyzer. The signal level is calculated by $V_{si} = V_{\mathrm{p}i} - V_{\mathrm{n}i}$, where $V_{\mathrm{p}i}$ is the level of the peak power at 2 kHz on the spectrum analyzer. Generally, the electrical levels $V_{\mathrm{p}i}$ and $V_{\mathrm{n}i}$ vary with the modes of experimental HG beams, and the electrical noise floor $V_{\mathrm{en}} = 60.61\,\mu\mathrm{V}$ is a constant value in our experiment. And the constant electrical noise floor $V_{\mathrm{en}} = 60.61\,\mu\mathrm{V}$ was detected without inputting light on the APD.

In **Supplementary Figure 7**, we illustrated the spectrums of detected optical power in APD with two different projection measurements and HG modes from 1 to 5. Moreover, we exerted an 1 V peak-to-peak level and 2 kHz

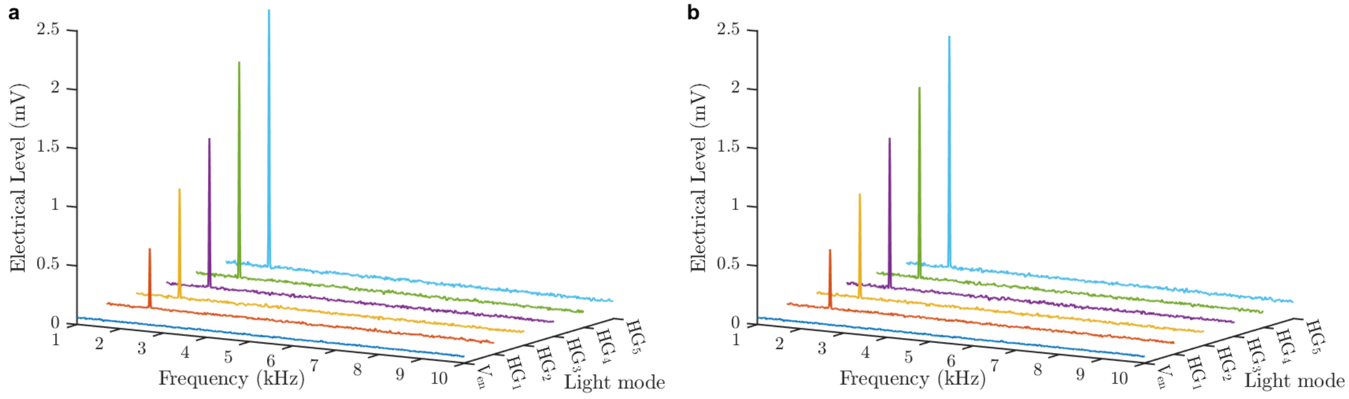

Supplementary Figure 7. Spectrums of detected optical power on APD of two different projection measurements with HG modes from 1 to 5. The first line in each figure is the noise floor without inputting light on the APD. The peak-to-peak level of driving signal on the PZT chips is 1 V, and the frequency of driving signal is 2 kHz. **a** Spectrums with projective measurement $\hat{\Pi}_1 = |u_{\hat{X}}^{\perp}\rangle\langle u_{\hat{X}}^{\perp}|$. **b** Spectrums with projective measurement $\hat{\Pi}_2 = |u_{\hat{P}}^{\perp}\rangle\langle u_{\hat{P}}^{\perp}|$.

Supplementary Table I. Detected electrical level of noise floor.

HG modes	Noise floor level[a]			Peak level at 2 kHz[b]	
	V_{en}[c]	V_{n1}	V_{n2}	V_{p1}	V_{p2}
HG_1		100.8 µV	99.18 µV	610.6 µV	603.4 µV
HG_2		113.4 µV	112.9 µV	1.043 mV	997.9 µV
HG_3	60.61 µV	122.7 µV	121.0 µV	1.399 mV	1.399 mV
HG_4		133.1 µV	130.1 µV	1.970 mV	1.756 mV
HG_5		142.9 µV	139.0 µV	2.339 mV	2.114 mV

[a] Measured from the corresponding spectrum.
[b] Measured from the level at 2 kHz in the spectrum analyzer, which corresponds to 1 V peak-to-peak level and 2 kHz driving signal on PZT chips.
[c] Corresponding to the electrical level of noise floor without light inputting in the APD, which is a constant value in the experiment.

driving signal on the PZT chips of the mirror in MZI. The measured noise floors and signal levels in the spectrum analyzer are illustrated in **Supplementary Table I**.

To determine the minimum detectable signal, we increased the driving voltages of PZT chips from 0 V to 0.5 V, the detected signal levels at 2 kHz of two projection measurements with HG modes from 1 to 5 are illustrated in FIg. 8. From the detected signal levels in spectrum analyzer, we can fit a linear relation between the driving voltage and the signal level at 2 kHz: $V_{\mathrm{s}i} = r_i V_{\mathrm{D}}$, where V_{D} is the driving voltage of PZT chips, and r_i is the linear coefficient calculated from **Supplementary Figure 8**. When $\mathrm{SNR}_i = 1$, $V_{\mathrm{s}i} = V_{\mathrm{sn}i}$. Therefore, the minimum detected value of parameters g_1 and g_2 in our experiment can be obtained by $\delta g_{1\mathrm{det}}^{\mathrm{min}} = g_1^{\mathrm{u}}(V_{\mathrm{n1}} - V_{\mathrm{en}})/r_1$ and $\delta g_{2\mathrm{det}}^{\mathrm{min}} = g_2^{\mathrm{u}}(V_{\mathrm{n2}} - V_{\mathrm{en}})/r_2$, where g_1^{u} and g_2^{u} are the unit signal amplitudes (which is given in the Method part of main text).

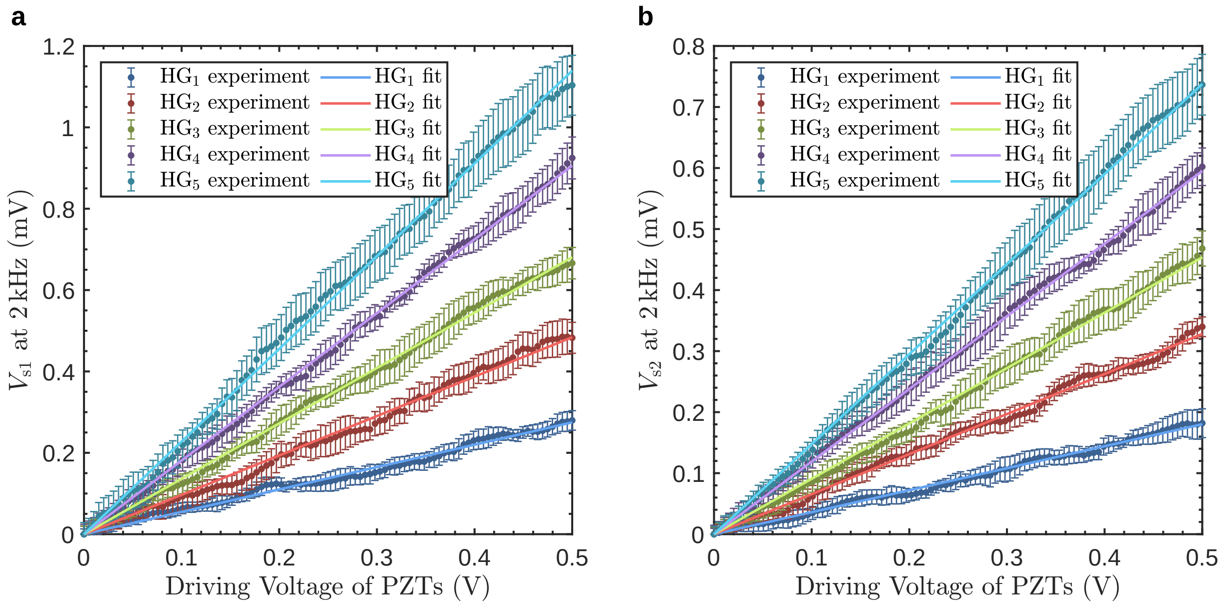

Supplementary Figure 8. Detected signal levels at 2 kHz of two projection measurements with HG modes from 1 to 5. The peak-to-peak level of driving signals on PZT chips increase from 0 V to 0.5 V. **a** Detected signal levels with projective measurement $\hat{\Pi}_1 = |u_{\hat{X}}^{\perp}\rangle\langle u_{\hat{X}}^{\perp}|$. **b** Detected signal levels with projective measurement $\hat{\Pi}_2 = |u_{\hat{P}}^{\perp}\rangle\langle u_{\hat{P}}^{\perp}|$.

[1] X.-M. Lu and X. Wang, Incorporating heisenberg's uncertainty principle into quantum multiparameter estimation, Phys. Rev. Lett. **126**, 120503 (2021).
[2] M. Ozawa, Uncertainty relations for joint measurements of noncommuting observables, Physics Letters A **320**, 367 (2004).

[3] C. Branciard, Error-tradeoff and error-disturbance relations for incompatible quantum measurements, Proceedings of the National Academy of Sciences **110**, 6742 (2013), https://www.pnas.org/doi/pdf/10.1073/pnas.1219331110.

[4] E. Arthurs and J. L. Kelly, B.s.t.j. briefs: On the simultaneous measurement of a pair of conjugate observables, The Bell System Technical Journal **44**, 725 (1965).

[5] J. Liu, H. Yuan, X.-M. Lu, and X. Wang, Quantum fisher information matrix and multiparameter estimation, Journal of Physics A: Mathematical and Theoretical **53**, 023001 (2019).

[6] B. Xia, J. Huang, C. Fang, H. Li, and G. Zeng, High-precision multiparameter weak measurement with hermite-gaussian pointer, Phys. Rev. Applied **13**, 034023 (2020).

[7] W. Kong, A. Sugita, and T. Taira, Generation of hermite–gaussian modes and vortex arrays based on two-dimensional gain distribution controlled microchip laser, Opt. Lett. **37**, 2661 (2012).

[8] S.-C. Chu, Y.-T. Chen, K.-F. Tsai, and K. Otsuka, Generation of high-order hermite-gaussian modes in end-pumped solid-state lasers for square vortex array laser beam generation, Opt. Express **20**, 7128 (2012).

[9] T. W. Clark, R. F. Offer, S. Franke-Arnold, A. S. Arnold, and N. Radwell, Comparison of beam generation techniques using a phase only spatial light modulator, Opt. Express **24**, 6249 (2016).

[10] O. S. Magaña Loaiza, M. Mirhosseini, B. Rodenburg, and R. W. Boyd, Amplification of angular rotations using weak measurements, Phys. Rev. Lett. **112**, 200401 (2014).
